# Application of telemedicine to improve access, and quality of healthcare services in Somalia: a perspective review and policy recommendations

**DOI:** 10.11604/pamj.2024.49.100.38299

**Published:** 2024-11-29

**Authors:** Bashiru Garba, Ali Olow Jimale

**Affiliations:** 1 Faculty of Medicine and Health Sciences, SIMAD University, Mogadishu, Somalia,; 2 Department of Veterinary Public Health and Preventive Medicine, Faculty of Veterinary Medicine, Usmanu Danfodiyo University Sokoto, Gidan Alkali, Nigeria,; 3 Faculty of Computing, SIMAD University, Mogadishu, Somalia

**Keywords:** Somalia, telemedicine, remote access, healthcare delivery, financial burden

## Abstract

Telemedicine is the use of virtual space to provide much-needed healthcare services to consumers, with the potential to revolutionize the delivery of healthcare. As the number of challenges facing Somalia continues to grow including the prolonged war, natural disasters, and migration of skilled healthcare professionals, the federal government may be overwhelmed and unable to provide sufficient basic healthcare to its citizens. This review was undertaken to highlight the current state of healthcare services delivery, the benefits of telemedicine, its potential to address the currently strained healthcare services, and the challenges that may hamper its adoption in Somalia. The review results show that the introduction of telemedicine into Somalia's healthcare will go a long way in supporting the efforts of the government to significantly improve the quality of healthcare by increasing accessibility and efficiency. The review also identified potential challenges that may hamper the adoption of this technology including policy barriers, hospital facility and information technology infrastructure, lack of trained healthcare workers as well as lack of awareness among the public on the benefits of telemedicine. Hence, given the current humanitarian situation facing the country and the determination to provide quality healthcare services at an affordable rate to its citizens, policymakers should view telemedicine as an attractive alternative to achieve these targets.

## Perspective

As the long-standing conflict in Somalia rages on, critical healthcare infrastructure and quality healthcare delivery have been severely hampered. Although considerable progress has been achieved in recent years in terms of improvement in the availability of healthcare services, the combined effect of the ongoing drought and continuing insecurity has largely eroded that achievement, leading to the displacement of rural pastoralist farmers from their settlements in search of food for themselves and their livestock. This humanitarian crisis has left the population weak, hungry, and susceptible to diseases. For many years, millions of Somalis especially those living in the rural areas and internally displaced camps found in major urban cities suffer from diseases due to malnutrition and lack of access to essential medicines [1]. Based on the Somalia National Development Plan 2020-2024, only 6000 health workers are estimated to provide services for the 15 million population. According to the World Health Organization (WHO), countries with less than 23 health workers per 10,000 populations will not be able to provide adequate coverage for primary healthcare. Unfortunately, based on the current estimates, there are only 4 health workers for every 10,000 Somalis, which indicates the acute shortage of healthcare workers in the country [2].

With the increasing population density, persistent conflicts, natural disasters, global economic challenges, and the frequent emergence of diseases and other public health emergencies, there is an urgent need for crisis-affected countries like Somalia to increase access and quality of healthcare services to its citizens.

Telemedicine refers to the practice of medicine using technology to deliver care at a distance. It is considered an efficient and cost-effective solution to fulfilling human´s basic health needs to facilitate healthcare delivery for patients in remote locations. It can also reduce the financial and logistic burden associated with traditional healthcare service delivery by reducing travel costs, and time while providing services to inaccessible locations due to insecurity [3]. There are a lot of success stories following the adoption of telemedicine in Europe, the Americas, and Southeast Asia [4]. However, Somalia has made limited attempts to incorporate telemedicine into its healthcare service. This paper presents a review of the current situation of healthcare services in the country, the problem of shortage of healthcare workforce, the potential benefits and urgent need of incorporating telemedicine into the healthcare delivery system as well as foreseeable challenges. The paper is organized as follows.

### General overview

**Telemedicine in conflict and crisis situations:** in most countries experiencing conflicts and other humanitarian crises situations, the government´s ability to deliver basic healthcare services is severely compromised. This is usually because of the destruction of government institutions including health infrastructure as well as a lack of accessibility to regions outside its control. The preservation of essential institutions and primary healthcare systems in crises and conflict situations is paramount. Unfortunately, in most conflict-affected countries like Afghanistan, South Sudan, Yemen, Syria, and Somalia, this has proven to be insurmountable [5]. Long-term crises severely stretch already weak health systems making it difficult for vulnerable populations such as women, children, and the elderly from accessing basic healthcare services [6]. This has resulted in countries impacted by fragility, natural disasters, and violence bearing the greatest burden of infectious diseases including HIV, tuberculosis, and malaria as well as higher maternal and child mortality [7,8].

As the number of conflicts, natural disasters, and violence continue to increase across the globe, so do the massive humanitarian situations leading to migration which is currently threatening global stability. Fighting this scourge has led to many innovations with eHealth among the most successful technologies that have shown great promise in addressing the epidemiological, geographical, and clinical disparities that characterize conflicts [9]. The ongoing COVID-19 pandemic has even expedited the interest and emphasized the need for the adoption of these digital technology platforms including telemedicine, the use of social media forums to enlighten the public about health hazards, as well as epidemiological mapping and tracing of disease events. Notable eHealth interventions in conflict-affected countries include Tele-Intensive Care Unit (Tele-ICU) in Syria, Telemedicine in Yemen and South Sudan, and Afghan telemedicine have added value in addressing the multitude of health challenges facing these countries [10]. However, despite the enhanced medical care that followed the implementation of these programs, the number of service provisions is grossly inadequate, and the service is not being accessed by most of the vulnerable population due to a lack of information and poor communication facilities.

**The situation of healthcare services and provision in Somalia:** despite the progress recorded in the healthcare service delivery in Somalia, a recent assessment by the United Nations Independent Expert about human rights in Somalia indicates that considerable efforts are still needed to ensure improvement in terms of coverage and quality of health service in the country with emphasis on the most disadvantaged areas [11]. The national health and demographic survey conducted in 2020 showed that morbidity and mortality trends have remained unchanged for several years, with the most common ailments the general population suffers being diarrhoea, acute respiratory infections, malaria, malnutrition, and other vaccine-preventable diseases [12]. At present, there are only a few government-owned public hospitals in the densely populated capital Mogadishu. This is because most of the health institutions were vandalized, and looted during the civil war, while a significant number of them have been taken over by internally displaced people. As a result, most people seek healthcare services from numerous private health facilities which are largely unregulated, expensive, and at the same time provide substandard services [13]. Because most of the population cannot afford such fees, they resort to self-medication and herbal medicines which are believed to be one of the main reasons why Somalia is among the countries with the highest maternal and child mortality rate in the world [14]. The limited healthcare services available in the country may not be unconnected with the long-standing humanitarian situation in Somalia driven by conflicts, insurgency, and a series of natural disasters including drought and famine which are the main factors responsible for the lagging health outcomes in the country. In addition to the impact on healthcare, the ongoing insecurity and armed conflict have made the country fragile, vulnerable, and poverty-stricken, thereby limiting opportunities for access to basic social services, including employment, housing, health, education, drinking water, sanitation facilities, and transportation among others.

Furthermore, the United Nations health expert in April 2022 warned that healthcare standards in Somalia are “dangerously low”. In the same vein, poor quality health services are thought to be among the factors holding back progress on improving health in countries at all income levels with resource-limited countries where a good number of hospitalized patients can expect to acquire an infection during their stay the most affected. Incidences of misdiagnosis, medication errors, indiscriminate antimicrobial prescriptions or inappropriate treatment, poor clinical facilities, and practices, as well as lack of adequate training and expertise, prevail in Somalia [15].

**Health workforce, and expertise:** studies revealed a gross shortage of health workforce in Somalia and the few ones available generally lack the required skills, knowledge, and resources necessary to execute their responsibilities as health professionals [16]. Shortage in the health workforce and scarcity of specialist health professionals are all features associated with humanitarian situations. This is because of the instability and security challenges, poor working conditions, low wages, as well as lack of professional development opportunities that characterize result in the migration of these essential human resources [17]. This assertion is supported by the reports of the 2006 World Health Report which showed that workers in the health system globally are experiencing increasing stress and insecurity among other complex arrays of challenges resulting in severe shortages, especially in conflict-affected countries. In that report, Somalia was among the 57 countries with a critical shortage of health service providers comprising medical doctors, nurses, and midwives [18]. Although mass migration of health workers is not only peculiar to humanitarian crisis countries like Somalia, many nations within the Eastern Mediterranean Region are also experiencing a similar trend due to increasing demand for skilled healthcare workers in developing countries who offer better pay and a safe, conducive working environment.

**Opportunities, and challenges in the implementation of telemedicine:** healthcare service within conflict regions like Somalia is complex owing to the entanglements between the general population, unrest, and deteriorating public services [9]. Telemedicine will certainly come in handy in addressing these challenges by increasing health delivery to affected populations. The provision of healthcare via telemedicine offers many advantages and has the potential to transform healthcare in Somalia by making specialty care more accessible to underserved rural and conflict-affected populations, as well as reducing financial burden in the form of transportation needs and costs [19]. Other potential benefits of telemedicine are improvement in the efficiency of therapeutic interventions, provision of psychological support as well as reducing cost and time [20]. The potential of telemedicine in providing significant improvement and cost-effective access to quality healthcare in conflict settings and the underserved communities in internally displaced persons (IDPs) and rural areas is considerable. All signs indicate a strong determination for the central government´s active participation in this venture. Telemedicine will bring benefits not only by providing healthcare services to the hard-to-reach areas and individuals residing in remote locations of the country, but it will also generate a new source of employment while subsidizing the cost of healthcare in the country. This remote service will also reduce the risks posed by the physical movement for both healthcare providers and patients, which will invariably reduce their financial burden.

With the current humanitarian situation in the country, telemedicine can also provide a mechanism to triage patients and mitigate the pressures on overburdened humanitarian response systems. Experience in other climes indicates that as conflicts persist, the situation worsens, hence the ability of telemedicine to maintain continuity of care for refugees, migrants, and internally displaced populations cannot be over-emphasized. Through remote access, patients in crisis-affected regions can be able to stay connected to the same providers having their medical records as they navigate this difficult situation. This sustained contact helps improve outcomes and health security for people and communities affected by conflict. Despite the identified potential benefits of telemedicine, the technology is also fraught with challenges and barriers associated with its implementation. In addition to the bureaucratic bottlenecks that characterize the introduction of telemedicine and its processes, the context of Somalia adds additional layers of complexity.

Some of the potential hurdles that must be addressed for the successful integration of telemedicine into Somali healthcare are highlighted here: a) Lack of health and eHealth literacy: the apparent inability of most of the Somali population especially those living in the rural areas and IDP settlements to find, access, and appraise digital health information to be able to make appropriate health decisions is a concern. A study conducted among Somali immigrants in Oslo showed that 71% of women lack health literacy [21]. The absence of digital competencies including high-speed bandwidth and application design have all been reported to constitute matters of concern that can potentially pose a significant challenge to the adoption of telemedicine in Somalia [22,23]; b) change in culture and behavior: telemedicine requires significant changes to the existing workflows and considering the complex nature of healthcare systems, this change in culture and behavior will be incredibly difficult. These changes need cuts across the board from the Somali government which will need to make a substantial financial commitment for installations and training of staff, to the nurses who will be required to provide additional or different care than they are used to, to the medical doctors at the linked facility that will be providing the diagnosis and treatment, as well as the client who may exhibit fear and distrust in the reliability and ability of the technology; c) confidentiality and privacy issues: studies have shown that issues of privacy constitute a significant barrier to the implementation of telemedicine [24,25]. Although Somalia currently does not have data protection legislation in force, matters of privacy could equally present a significant hurdle in the deployment of telemedicine particularly because Somalis by the nature of their culture and beliefs place considerable importance on gender concordance with healthcare service. Also, the possible lack of understanding of the security of their data can make them resist its adoption; d) infrastructure: other important considerations are because this virtual system requires synchronized information exchange, videoconferencing, or real-time telephone conversations, its deployment in conflict-affected settings may be challenging. This can be attributed to insufficient telecommunication infrastructures, shortages of trained healthcare personnel, and healthcare insecurity, among others [26].

Despite, these challenges the prospects of telemedicine implementation in Somalia look promising. Some of these opportunities are: a) Technological adaptation: a good instance is a rate at which the Somali community has adopted information technology into their daily lives like the cashless banking policy that is largely popular even among rural dwellers. Another good example is Hello! Caafi, a Somali women-led telehealth service provider in partnership with Response Innovation Lab (RIL) has made a considerable effort by delivering healthcare, health education, and health information services via remote technologies to 70 different rural communities in the country (Response Innovation Lab, 2021). Their services also give priority to women of reproductive age due to their increased vulnerabilities as mothers and breadwinners in most households. The organization leveraged the fact that the majority of rural Somalia has low population density and familiarity with digital technology as exemplified by Somalia´s cashless economy, whereby even people in rural areas are familiar with mobile money transfer services among other technological advancements. According to the World Bank report, the development of information technology in Somalia is adjudged to be one of the major success stories in the country's efforts to rebuild and ensure productivity among its citizens [27]. If the country continues its path by harnessing the power of digital technology and its deployment to promote health and wellness, this will go a long way in providing increased access to high-quality health services; b) Somali diaspora: leveraging on its large pool of highly skilled diaspora, Somalia can use telemedicine to facilitate healthcare delivery for patients in remote locations. From time immemorial, the Somali diaspora that cuts across three generations has contributed immensely to the progress and development of the country through the transfer of expertise, investments, and political influence. A good example is the “HIRDA” which stands for Himilo Relief and Development Association. The organization was founded in the year 1998 by members of the Somali diaspora residing in the Netherlands. The organization´s primary objective was to support Somalia and help the government and the people address the many challenges facing the country including poverty, and health. From 2008 to 2011, the organization was able to train 279 social health workers that run mother and child health centers, immunization campaigns, and health posts in rural areas, all established by the organization [28]; c) IT workforce: our local universities are producing many IT workforces capable of developing telemedicine information systems and installing the required technologies such as cameras. All these engineers can contribute significantly to Somalia's telemedicine implementation roadmap. There are also Somali IT experts and computer scientists living both inside and outside of Somalia who can contribute to telemedicine initiatives; d) stakeholder interest: recent efforts by some international donors and the commitment shown by the Somali government to adopt and implement telemedicine in Somalia are encouraging. For example, the International Organization for Migration (IOM) is championing the deployment of telemedicine into the Somalia healthcare system by equipping and strengthening the existing healthcare institutions in the country with digital technology to enhance their capacity to provide healthcare services through remote diagnosis and treatment. For the first time in over thirty years, the World Bank under the umbrella of its International Development Assistance (IDA) grant in collaboration with the Global Financing Facility for Women, Children, and Adolescents has approved an investment package worth USD 100 million into the Somalia healthcare system [29]. All these interests could contribute to the successful implementation of telemedicine in Somalia.

**Framework for telemedicine implementation:** traditionally, a telemedicine program combines several technological tools under a common system to expand and enhance the delivery of healthcare services to patients. It should be able to create an environment and opportunity where healthcare providers and patients interact in synchrony. In this way, the patients can receive specialized and efficient care in a group or individually as well as other follow-up consultations if necessary. The program should be designed for integration into the broader healthcare services in the community and make provisions for adequate funding and quality improvement.

The telemedicine implementation framework is intended to guide health systems and providers in navigating the complexities associated with its implementation, especially in conflict-affected countries.

**Preparatory stage:** this initial phase entails undertaking a variety of assessments regarding the required skills, capabilities, and operating environment, including the beneficiary community. In addition, research efforts, consultations with relevant stakeholders to identify gaps, and the development of a viable business plan are paramount for the successful implementation of telemedicine practices. Subsequently, factors such as patient locations, health conditions to be treated, and providers' delivery care that can affect the operational and clinical workflows are addressed.

**Development stage:** this is the phase where the actual building of the telemedicine service commences, including developing a standardized clinical workflow and operational protocols. In this stage, basic infrastructures are installed and factors that can affect workflows such as registration, scheduling, billing and format of payment, communication outlets, and clinical documentation are put in place. Also at this stage, responsibilities are assigned and each individual accountable for the operation of each step is identified.

**Implementation:** the stage encompasses the period of training, familiarization, testing, and practice. Relevant healthcare workers are educated on the routine operations of the program and workflows. They are trained on how to handle and operate the equipment, make, and receive calls, and practice.

**Operations phase:** at this point, in consultation with major stakeholders, an evaluation is undertaken to align the delivery strategy and operations. The aim is to ensure the delivery of a credible and high-quality service efficiently. This should be done with a focus on the beneficiaries, as well as the process, and performance of the telehealth services to drive operational efficiency.

**Quality control:** for the sustainability of the telemedicine program, continual quality improvement to provide a formal mechanism that monitors the activities, operations, and delivery services must be put in place in alignment with the evolving needs of the customer. This can be achieved via routine assessment of the key performance indicators and institution of process improvement tools. Other important considerations are: 1) facilities: the deployment of telemedicine facilities in conflict-affected settings like Somalia will require several resources ranging from physical space for installation of the equipment and videoconferencing or consultation room. Among the common technological installations necessary for conflict-affected settings like Somalia are videoconferencing facilities, telemedicine carts equipped with cameras and screens, and remote patient monitoring devices [30]. The type of installations to be put in place will also depend on the type and nature of services needed. This information can be obtained from international partners like the National Telehealth Technology Assessment Resource Centre. These organizations can assist in guiding the implementation, expansion, and sustenance of telehealth services; 2) human resources: others are the employment of trained staff to man the facility, technological installations, and the establishment of partnerships that will provide expert consultations and guidance, especially in areas where specialists are lacking. Areas of priority in Somalia that can benefit from telemedicine consultation are neonatal intensive care, cardiovascular care, as well as tele-education which will allow local medical professionals to access a growing online, digital, and video-based reference “library” of diagnostic expertise within Somalia and beyond its borders. Other important stakeholders that can be affiliated are academic medical centers or larger regional hospitals that can provide access to specialty care services through telehealth.

## Conclusion

Telemedicine can´t address all the healthcare challenges Somalia is facing now. Notwithstanding, remote care can play a critical role in crisis response, as well as addressing the shortage of healthcare workers and specialties that are lacking in the country. The lack of comprehensive policies and regulatory frameworks necessary for enhanced security, privacy, and confidentiality of the patient data shared electronically can be addressed by engaging relevant government authorities for necessary legislation that will ensure that both patients and physicians are protected. One important measure to address this problem will be to provide patients with informed consent forms that explain their rights before subjecting them to the services. Also, ensuring that measures are put in place to address issues of accountability and clearly spelt-out penalties for the unauthorized collection, and usage of electronic patients´ data. On the other hand, barriers like the availability of healthcare facilities, lack of trained IT technicians, and IT infrastructures including internet connectivity, electricity, and computer hardware can be tackled with a sound financial investment and training of personnel on the technicalities associated with the operations of telemedicine devices. The current reality is that healthcare systems in Somalia are collapsing under the immense pressure of humanitarian crises, such that the existing healthcare systems can't serve vulnerable populations effectively. Hence, despite the identified bottlenecks, the prospect of telemedicine contributing to better healthcare in Somalia is considerable.
